# Acoustic and auditory relation of creak/creaky settings in telephone call and direct recording samples: An approach in the light of forensic phonetics

**DOI:** 10.1371/journal.pone.0346033

**Published:** 2026-04-21

**Authors:** Sara Gomes Rosa, Sabrina Bravo Baglioni, Renata Regina Passetti, Ana Carolina Constantini

**Affiliations:** 1 School of Medical Sciences, Universidade Estadual de Campinas, Campinas, São Paulo, Brazil; 2 Postgraduate Program in Linguistics, Federal University of São Carlos, São Carlos, São Paulo, Brazil; Osaka University, JAPAN

## Abstract

This study assessed the possible relationship between acoustic and auditory analyses of vocal samples extracted from telephone calls and direct recordings. Samples from a database of auditory assessments of Voice Quality (VQ) performed by experienced phoneticians using Vocal Profile Analysis were used. Analyses were made of recordings of spontaneous speech samples from five men (aged 20–56, diagnosed with dysphonia), obtained simultaneously by telephone calls and direct recordings. Acoustic analysis was conducted by extracting 20 melodic measurements and VQ descriptors, in an automated manner using a script in the PRAAT software. These measurements were then compared with the results of database analyses using descriptive statistics. The results showed that, despite the differences obtained between the recording channels, acoustic parameters associated with voice quality, such as jitter, shimmer, and harmonic-to-noise, presented consistent correlations with perceptual judgments of non-modal phonation, especially in segments with creak or creaky voice. The analysis revealed that the telephone channel tended to smooth out abrupt peaks in the acoustic measurements, but did not compromise the identification of predominant voice quality patterns. This stability suggests that, even with the inherent signal degradation in telephone transmission, automated acoustic analyses can provide relevant and reliable information for forensic phonetic analysis. We conclude that the integration of acoustic and perceptual analyses, even using recordings from different channels, is feasible and valuable for the forensic assessment of voice quality. These findings highlight the importance of a careful methodological design that considers the particularities of the recording channels and reinforce the potential of forensic phonetics to provide robust evidence in legal contexts.

## Introduction

This study addresses the interdisciplinary field of forensic phonetics (FP), whose purpose is to identify a speaker based on their vocal characteristics [1]. In this field, several activities are conducted in order to answer questions related to phonetic issues in legal proceedings [2].

Unlike traditional experimental phonetics, which focuses on hypothesis testing under controlled conditions, forensic phonetics treats speech data as evidential material in legal contexts and predominantly deals with material produced in natural, uncontrolled contexts, such as spontaneous conversations and telephone recordings. In these situations, the recording channel, signal quality, and communicative context impose relevant methodological constraints, requiring descriptive approaches and cautious interpretations focused on individual speaker behavior and intra-speaker variability rather than broad population-level generalizations [3].

One of the common tasks in this field is Forensic Speaker Comparison examination, which looks for variations between compared speech samples to determine whether two audio recordings originate from the same individual [4]. Phonetics can be analyzed from three perspectives: articulatory, perceptual, and acoustic [5]. In forensic practice, an expert determines which procedures to use to compare the samples. One of the most frequent models is a combination of acoustic and perceptual analyses [6].

Analyses based primarily on voice quality (VQ) are relevant in investigations of this nature, as it constitutes a promising indicator of speaker identification. Such analyses can provide, among other data, information about the speaker’s linguistic identity and the linguistic community to which the speaker belongs [7].

Regarding the perceptual evaluation of voice quality (VQ), the Vocal Profile Analysis (VPA), developed by Laver (1980) [8] to describe a speaker’s vocal profile, has also proven applicable to the forensic context, precisely because it incorporates detailed and discriminative perceptual aspects [9].

Among the parameters that compose the VPA are several settings that reflect specific voicing types, such as creak and creaky sounds. The non-modal phonation creak corresponds to an irregular vibration of the vocal folds at a low frequency, resulting from strong glottal adduction. In contrast, creaky voice refers to a voice quality feature, rather than a total phonation type, characterized by laryngeal constriction and periodic irregularity, which may include, but is not limited to, the creak setting [8,10].

Researchers describe creaky as a perceptual category that encompasses creak as its most extreme point [11].

Although such characteristics are not frequently the focus of forensic analyses, their investigation becomes relevant in this study because they represent perceptual VQ data potentially useful for distinguishing between speakers.

With respect to the application of the VPA in the forensic setting, the literature indicates that when the evaluator is properly trained, the protocol is promising for constructing vocal profiles in lawsuits [12]. Furthermore, its relevance is evident not only in forensic practice but also in academic research, as this system is preferred by forensic phoneticians when compared to other perceptual analysis methods [13].

Regarding acoustic analysis, there is a consensus that it is a VQ analysis method with the potential to complement perceptual assessment [14]. Its application has numerous advantages, including the ability to convert sound signals into numerical data, making speech a quantifiable object [15].

However, acoustic analysis has more challenges in practice, as it is not always possible to perform it. Measurement extraction in forensic practice can be affected by factors that can compromise its results, such as voice overlapping and background noise [16]. Common challenges in the field, such as recordings from wiretapping, also complicate acoustic analysis [17].

The analysis of audio extracted from telephone channels is challenging due to the various effects of telephone transmission that can affect the extraction of acoustic speech measurements [18–22].

The impact is not limited to the acoustic aspect, as reported by Passetti and Constantini (2019) [23]: the effect of the telephone can also influence the correct assessment through auditory perception when creating a speaker’s vocal profile. Signal loss, ambient noise, and distortion of the telephone itself also contribute to the complexity of this type of analysis when comparing speakers [24].

The literature has limited studies assessing the impact of telephone transmission on measurements related to voice quality, especially combining perceptual and acoustic analyses. This differentiates our study and makes it relevant to the scientific community.

Recent studies have also investigated the impact of telephone transmission on acoustic measures of voice quality, such as the work presented at Interspeech 2024, which compares acoustic parameters between high-quality recordings and VoIP telephone speech [25]. However, these studies differ from the present work by adopting an experimental and technology-oriented framework, focusing primarily on transmission types and isolated acoustic measures. In contrast, the present study is grounded in forensic phonetics and examines semi-spontaneous speech produced under natural conditions, integrating automated acoustic measures with specialized auditory judgments, an aspect that is central to the interpretation of vocal evidence in forensic contexts.

The guiding question of this study refers to assessing how voice quality analysis, through the extraction of acoustic measurements and their relationship with perceptual judgment, can be used in different situations involving telephone calls and direct recordings.

Therefore, this study aimed to investigate the possible relationship between the acoustic and perceptual analyses of vocal samples extracted from telephone calls and direct recordings, confirming the relationship between phonatory settings and acoustic measurements.

## Materials and methods

This is a descriptive, retrospective, and quantitative study that compared the extraction of perceptual measurements from a database with analyses of acoustic measurements. The study was approved by the Research Ethics Committee of the Universidade Estadual de Campinas (UNICAMP) opinion no. 4.895.873.

### Database

This study used samples of telephone calls (TC) and direct recordings (DR) from a database created in a previous study [23], which included the stages of subject selection, sample collection, and perceptual evaluation. These stages are detailed later.

The data were accessed for research purposes after approval by the Ethics Committee on August 10, 2021. Because the present study addressed a different research objective, the Research Ethics Committee required recontacting the original participants to obtain new written informed consent authorizing secondary use of their recordings; this procedure was realized after approval by the Ethics Committee too.

The database contained voice recordings of eight men aged 20–56 years. To observe how different components of VQ behaved under the effects of telephone transmission, all subjects had an otorhinolaryngological and speech-hearing pathology diagnosis of functional or organofunctional dysphonia. At the time of recording, they were being treated at the Speech-Language Pathology Voice Clinic of the Centro de Estudos e Pesquisas em Reabilitação Gabriel Porto, UNICAMP, Brazil.

Data collection was conducted simultaneously by direct recording (DR) and telephone call (TC). In DR, a Yoga HM20 headset microphone (Yoga Electronics Co., Taipei, Taiwan) was used, connected to a Zoom H4N digital recorder (Zoom North America, Hauppauge, New York, USA). In LT, a Nokia 110 receiver telephone (Nokia, Espoo, Helsinki, Finland) and an iPhone 5s calling telephone (Apple Inc., Cupertino, California, USA) were used, connected to a Behringer UCA222 sound card (MUSIC Tribal Global, Makati City, Metro Manila, Philippines) and a Dell notebook (Dell Inc., Hopkinton, Massachusetts, USA).

The recordings were made in WAV (WAVE audio format) and stored in Audacity 1.2.6 software. The sampling rate was 44.1 kHz and 16-bit quantization. The simultaneous recording of the samples was performed in a soundproof booth, in a semi-spontaneous context of informal conversations with the interviewer about daily life activities such as work and hobbies. Consequently, both recordings contained exactly the same linguistic content, as well as identical prosodic and segmental-acoustic information, differing only in the effects of telephone transmission.

After data collection according to Passetti and Constantini (2019) [23], a perceptual experiment was conducted to determine the vocal profile of each speaker using the Vocal Profile Analysis (VPA) script (Laver, 1980), in its Brazilian Portuguese version [26].

At that time [23], both the DR and the TC were assessed using sample excerpts containing about 20-second stimuli by six phoneticians with different backgrounds who had received prior training in VPA. In our study, data obtained from the previous study were incorporated in this task, correlating their auditory perception findings with the extraction and analysis of acoustic measurements.

For further details on how the perceptual judgment using the VPA was conducted, see the study from which these data originate, Passetti and Constantini (2019) [23].

### Sample handling

Vocal samples from five subjects were suitable for use according to the procedural recommendations of the institution’s Ethics Committee for conducting a second experiment, as all subjects should be contacted again and sign a new informed consent form. However, only five subjects were successfully contacted, representing the total number of the current sample.

In the initial stage, audio was processed using PRAAT 6.1.38 and converted to the mono channel format using the software tools. For each subject, two speech samples were analyzed, corresponding to different recording types: one from TC and one from DR, totaling 10 recordings evaluated (5 TC x 5 DR).

The segmentation and labeling steps were performed in a single annotation layer in PRAAT, delimiting the complete speech segments separated by silent pauses. The procedure was performed on the five DR samples and only replicated for the TC samples, since the sample pair for each subject had exactly the same content.

For the present study, we chose to analyze each sample in its entirety, from the moment the participant answered the phone call until the end of the conversation. This methodological decision differs from the previous study, which used 20-second excerpts following VPA recommendations.

The net durations of the samples ranged from 4 minutes 27 seconds to 7 minutes 12 seconds, with a mean duration of 6 minutes 15 seconds. These durations correspond exclusively to the speakers’ own speech and do not include the interviewer’s speaking turns, as only the participants’ vocal productions were considered for segmentation and acoustic measure extraction.

Despite this temporal variation, no impact on the perceptual or acoustic results is expected, as VPA guidelines indicate that approximately 20 seconds of speech are sufficient to characterize a speaker’s voice quality.

Regarding the acoustic analysis, parameters were extracted for each segment between silent pauses, but only the global mean values of each measure were used for the statistical analyses. Thus, each speaker is represented by aggregated values summarizing the acoustic behavior across the entire speech sample, minimizing any influence of duration differences between recordings. In this sense, using the full recordings maximized the available linguistic material without introducing bias related to sample length variability.

### Extraction of acoustic measurements

A set of 20 acoustic measurements were automatically extracted from each speech excerpt segmented into 10 samples using the Prosody Descriptor Extractor script (Barbosa, 2020), as follows: median fundamental frequency (f0med), f0 standard deviation (f0sd), f0 interquartile semi-amplitude (f0SAQ), minimum f0 (f0min), maximum f0 (f0max), standard deviation of maximum f0 (sdf0peak), mean bandwidth of f0 peaks (f0peakwidth), smoothed f0 peak rate (f0peak_rate), standard deviation of f0 peak positions (sdtf0peak), mean of the 1st positive derivative of f0 (df0posmean), mean of the 1st negative derivative of f0 (df0negmean), standard deviation of the 1st positive derivative of f0 (df0sdpos), standard deviation of the 1st negative derivative of f0 (df0sdneg), spectral emphasis (emph), intensity variation quotient (cvint), long-term spectral slope between the 0–1000 Hz and 1000/4000 Hz bands (slLTASmed), harmonic-to-noise ratio (hnr), soft phonation index (SPI), local shimmer (shimmer), and local jitter (jitter).

### Relation between acoustic measurements and perceptual data

It was proposed, for the cross-analysis of the different approaches, to consider from the perceptual stage [23], the judges’ evaluations referring to the specific VQ section of the VPA protocol: the topic entitled “Phonatory Features”, which includes the assessment of voicing type — voice neutral, falsetto, creak and creaky; laryngeal friction — whisper and whispery; and laryngeal irregularity — harsh voice.

However, it was only possible to include in the present analysis the VPA judgments related to the voicing type — voice neutral, creak or creaky voice. Evaluations indicating the presence of falsetto were not perceived by the judges in the previous stage. The presence of laryngeal friction and laryngeal irregularity was excluded from the analysis due to the low number of occurrences, which hindered statistical comparison.

After an in-depth reflection on the data, the VPA judgments were categorized as modal or non-modal, the latter referring to the perception of creak or creaky voice settings by the judges.

The respective results of the perceptual analysis, as well as the extracted acoustic measures, were organized into tables considering the judgment of modal or non-modal voice, corresponding to the Voicing Types topic in the VPA.

To analyze the relationship between the judges’ perceptual assessments and the extraction of acoustic variables, the datasets were cross-referenced. A spreadsheet was created in Excel containing ten perceptual judgment entries (5 speakers × 2 channels), as well as the mean values for each acoustic measure, for each speaker and channel (DR or TC), resulting in a total of 275 data points and 26 variables.

Additionally, the spreadsheet contained a single variable derived from the VPA data — the Non-Modal Rating (NMR), a proportional count variable indicating how many of the six judges rated the speaker in that channel as having non-modal phonation.

To better illustrate the results, data were organized in Excel, producing a frequency graph of the non-modal judgment and a heat map with the acoustic values. Acoustic scatter plots were also created for each measurement in relation to non-modal voice perception.

## Results

This section presents a descriptive and exploratory analysis of the findings, correlating the acoustic measurements of the vocal samples with the perceptual judgments of non-modal phonation (creak and creaky voice). The analysis considered ten vocal samples from five subjects, recorded simultaneously on two channels, and the perceptual evaluation of six phoneticians.

For each subject and channel, the mean of each acoustic measurement was calculated, as well as the absolute frequency of the number of judges who attributed a perception of non-modal phonation to that sample.

## Perception of non-modal settings

**Fig 1:**
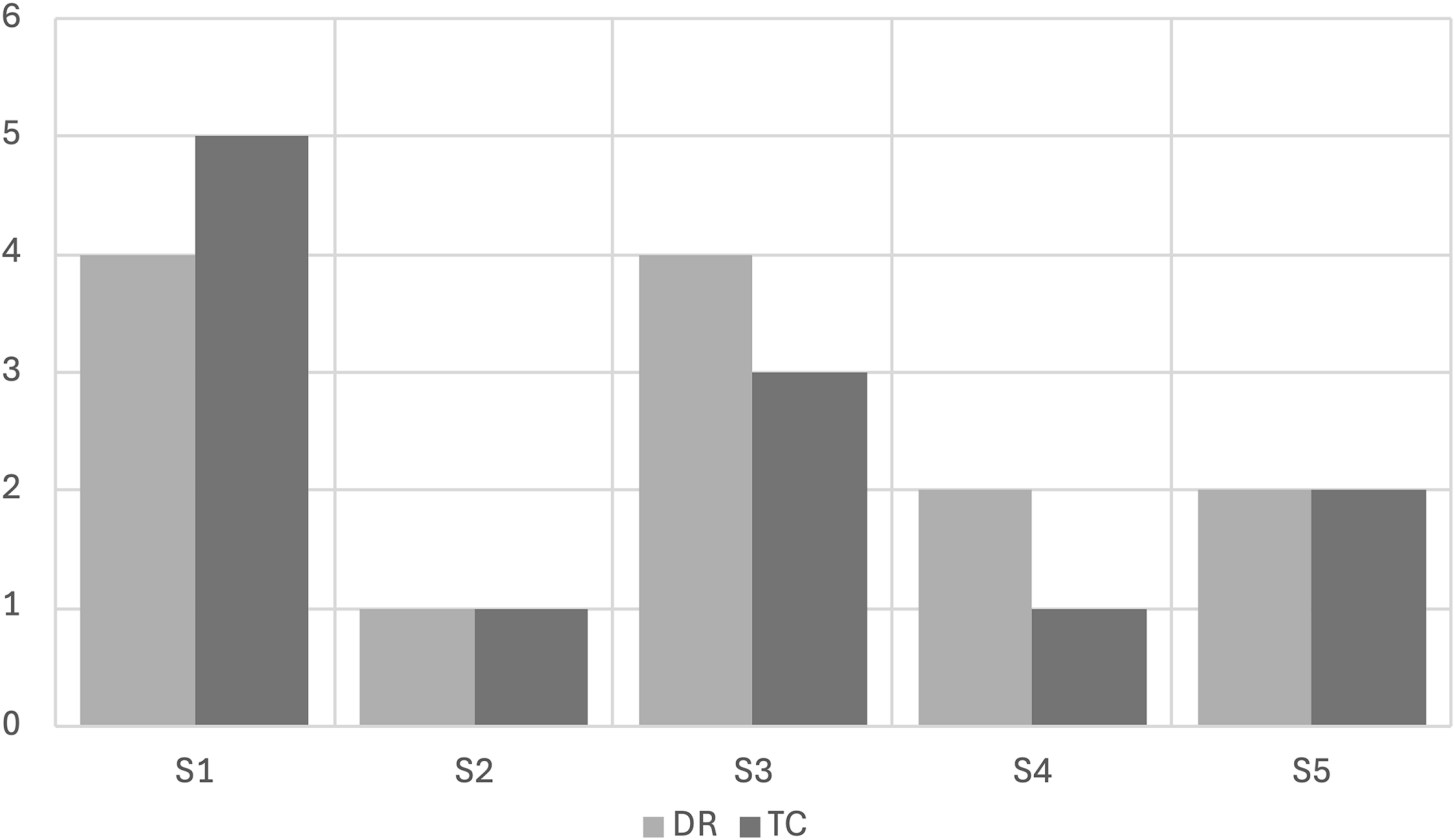
Judges’ assessment of voice Non-Modal Rating (NMR) per subject in direct recording and telephone call samples. Legend: DR – Direct Recording; TC – Telephone Call.

Fig 1 shows how many of the six judges classified phonation as non-modal (creak or creaky) for each subject-channel combination.

Perception varied between channels and subjects, with values ranging from 1 to 5 judges. Fig 1. shows that the channel had an impact on the judge’s auditory perception, making it uneven for three subjects – S1, S3, and S4 – who presented different results when comparing the two recording modalities. For S2 and S5, the judge’s perception of creak or creaky voice did not differ between DR and TC.

In general, for the study group, the samples derived from direct recording were judged as non-modal more frequently than those from telephone calls. Only for S1, TC showed a higher frequency of non-modal judgments, which may be a speaker effect. Therefore, a varied effect occurred, since this trend was not uniform for all subjects.

S1 obtained a high perception of non-modal setting in both channels (4 and 5), while S2 maintained a minimal value in both (n = 1). S3, S4, and S5 showed more significant variations in perception (4 and 3, 2 and 1, and 2, respectively), indicating that the channel may influence each vocal profile differently. Analysis in the forensic context must be detailed and consider the effects of both the subject and the channel.

The frequency graph was also used to explore possible relationships between the acoustic data and the auditory judgments, which will be presented below.

### Relationship between perceptual judgment and acoustic measurements

**Fig 2:**
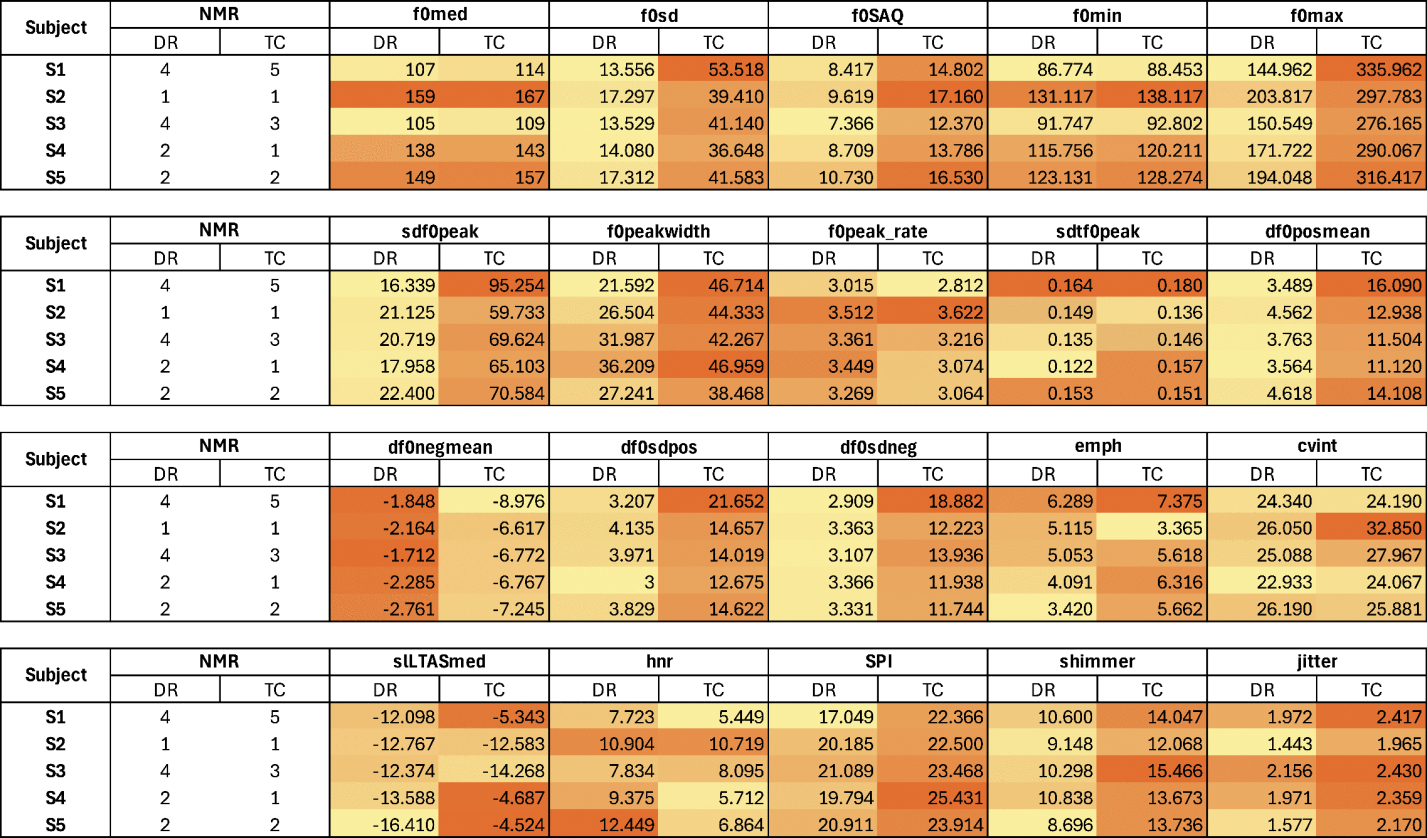
NMR assessments and acoustic variation heatmap across channels and subjects. Legend: DR – Direct Recording; TC – Telephone Call.

To highlight which measurements increase or decrease according to Non-modal Judgment, Fig 2. the charts present heat maps with a color scale that compare variations between channels and subjects for each acoustic measurement. The cells change their color according to the measurement value: the closer to yellow, the lower the value; the closer to orange, the higher the acoustic value among the 10 values extracted for the respective parameter.

The behaviors observed suggest possible associations between the perception of non-modal phonation and certain acoustic patterns.

Measurements related to fundamental frequency variability, such as standard deviation and interquartile semi-amplitude, tended to increase in most measures per subject as the NMR increased. This pattern suggests that voices perceived as non-modal may be associated with greater f0 variability, reflecting more irregular production patterns, such as those expected in creaky or creak settings. Regarding the median, subjects with higher NMR tended to have lower values.

Regarding the derived measurements of f0 peaks, an almost consistent variation was observed in their values in the TC for all subjects, making it difficult to identify patterns related to NMR. In DR, the drops in melodic contour, demonstrated by a more negative mean of the negative derivative, seem to be aligned with the perception of creak or creaky voice, particularly in subjects with high NMR.

The measurements of VQ shimmer, jitter, and soft phonation index tended to increase for most subjects as non-modal perception increased. On the other hand, long-term spectral slope and spectral emphasis showed less linear trends, but still indicated significant variations between samples with different non-modal voice scores.

Specifically regarding spectral emphasis, voices less judged as non-modal seem to have higher values, possibly reflecting a clearer and more efficient articulation pattern. However, there is no clear trend because the variation between channels is significant.

The harmonic-to-noise ratio, a measurement of the harmonic stability of the signal, showed a tendency to decrease as the NMR increased. This relationship points to a relative increase in glottal noise in samples perceived as non-modal, especially in direct recording, where the signal presents a high level of spectral richness.

In addition to the behaviors described above, subjects with higher NMR, such as S1 and S3, also presented the lowest f0 median values and the highest values of standard deviation, peak standard deviation, shimmer, and jitter. Subjects with minimal judgment, such as S2, maintained lower values in instability measurements and higher values in measurements such as harmonic-to-noise ratio and spectral emphasis.

The patterns observed in both DR and TC samples support the idea that certain acoustic features align, although partially, with the perceptual judgments made by the judges. Also, data suggest that the recording channel may influence the acoustic measurement of certain phonatory settings or their perceptual detection, but this influence is not systematic among the subjects.

**Fig 3:**
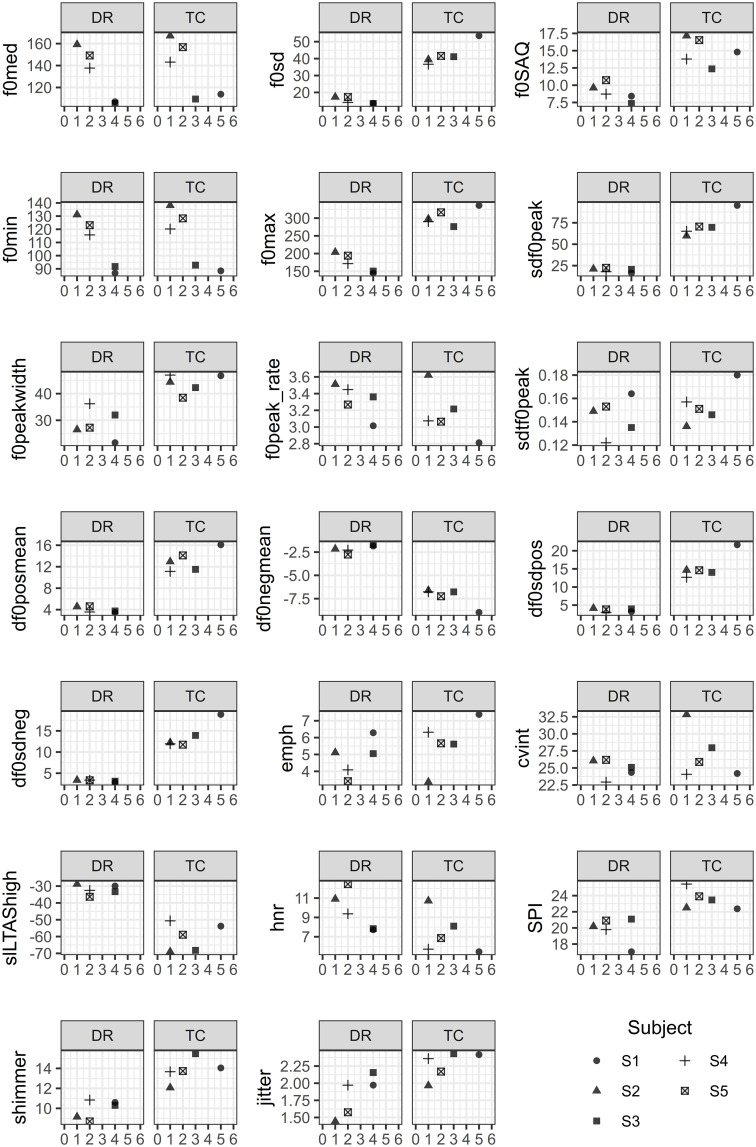
Dispersion of mean acoustic values as a function of NMR, by channel and by subject. Legend: DR – Direct Recording; TC – Telephone Call.

Fig 3 shows the dispersion of acoustic measurements in relation to non-modal voice perception. Each point in each figure represents the mean acoustic measurement per subject in a channel. The X-axis shows the number of judges who evaluated the phonation as non-modal (creak or creaky), and the Y-axis shows the mean value of the acoustic measurement.

In general, increasing or decreasing patterns are observed between the measurements and non-modal judgments, but with inter-subject variations.

Subjects with higher NMR (such as S1 and S3) tend to cluster their points in regions with higher values of instability measurements (shimmer, jitter, sdf0peak) and lower HNR values. Subjects with lower NMR (such as S2 and S5) concentrate their points in regions with less glottal instability and higher periodicity, indicated by higher HNR values and lower jitter/shimmer values.

These patterns are largely maintained in both channels, but with some significant differences between DR and TC. In several cases, the points in the TC channel show greater dispersion even for the same NMR, as well as a tendency toward flattening of the extreme measurements, suggesting a possible effect of telephony compression on the acoustic parameters.

### Inter-subject variation

Individual subject analysis showed that the relationship between acoustic measurements and non-modal perception scores is not standardized in all cases. For S1 and S3, an alignment is observed between perceptual enhancement and intensification of acoustic measurements of instability (such as jitter, shimmer, and sdf0peak), while S2 and S5 presented more stable patterns and low non-modal perception scores in both channels.

This inter-subject variation reinforces the need for approaches that consider the individual vocal profile, in both the acoustic analysis and perceptual interpretation. Also, it highlights the relevance of using methodologies that combine different types of analyses, especially in forensic contexts where decisions are made on a case-by-case basis.

## Discussion

This study aimed to investigate the possible relationship between acoustic and perceptual analyses of voice quality in samples simultaneously extracted from telephone calls and direct recordings. This approach sought to fill a gap in the literature by combining perceptual judgments and acoustic measurements in different recording channels.

This study offers a relevant methodological contribution to the field, while also reinforcing the role of forensic phonetics in the critical analysis of voice evidence. The originality of the proposal lies exactly in this articulation between human perceptions and automated metrics under different technical conditions, suggesting ways to improve the interpretation of vocal data in forensic contexts.

The exploratory analysis revealed relevant patterns in the behavior of acoustic measurements in different recording channels and their relationship with perceptual judgments of non-modal phonation (creak/creaky voice).

The findings are discussed considering the literature on forensic phonetics and acoustic phonetics, with an emphasis on the methodological and interpretative implications for the Speaker Comparison examination.

Regarding the influence of the recording channel, a central aspect of this discussion, the effects of telephone transmission were observed in several measurements, reinforcing the need for a contextualized reading of these data.

It should be noted that, although the audio samples were collected simultaneously on both channels (DR and TC), the TC samples underwent compression and band-clipping. This technical characteristic directly influences both auditory perceptions [23], by eliminating higher-frequency information and reducing spectral signals, and the extracted acoustic measurements [20], which reflect a signal with less harmonic content and more susceptible to artifacts. This is especially relevant in the forensic context, in which a significant portion of the samples analyzed come from telephone records [1,6].

Therefore, the difference between the channels mentioned here does not simply represent a change in the recording medium, but a structural change in the speech signal, which explains the variation in NMR scores and acoustic measurements between DR and TC.

The central and dispersion measurements of f0 were consistently lower in the telephone channel, in agreement with previous studies on bandwidth limitations in the compressed digital channel [6,27]. This is particularly important given that f0 is often a key parameter in comparative voice analysis [20,28], although its stability across recording contexts is questionable [29,30].

Also, measurements related to the structure of f0 peaks, such as mean peak width, frequency of occurrence, and standard deviation of peak positions, showed significant differences between the channels. Such changes can be interpreted as a reflection of signal degradation, which affects both the perception and the automatic extraction of f0 contours [6,29].

The literature indicates that creak voices often present abrupt and poorly defined peaks, with irregular temporal organization [31,32]. This description is consistent with the findings obtained and reinforces the idea that temporal f0 patterns (not just centrality measurements) are essential for the perceptual characterization of non-modal phonation [30].

Studies indicate that the telephone transmission system causes significant losses in acoustic information that is relevant to voice quality analysis [14,20]. For some acoustic variables traditionally associated with voice quality, such as the soft phonation index, shimmer, and jitter, despite showing consistent correlations with the perceptual variable NMR in this study, also showed differences between channels.

In addition, it is equally evident that some measurements of this nature remain stable enough to reflect perceptual patterns, as observed in the correlation between the harmonic-to-noise ratio and the intensity variation coefficient with NMR, also in TC.

The results confirm that non-modal voice perception, measured here by NMR, is strongly associated with acoustic measurements that indicate temporal and spectral voice instability (especially jitter, f0 variations, and intensity variation). f0 measurements such as median or maximum value, although useful for global voice characterization, proved to be of little relevance for perceptual purposes of voice quality. Also, the differences between the channels suggest that the telephone channel may mask some crucial acoustic cues, reinforcing the need for caution when pairing acoustic and perceptual analyses in the forensic context, as previously discussed by Davidson (2021) [30] and Klug et al. (2024) [33].

The atypical NMR of S1, unlike the others that showed divergence in the channel comparison, was the only one with a higher number of non-modal phonation judgments in the telephone channel. This finding differs from the general trend observed and illustrates the importance of considering individual vocal profiles in forensic analyses [30,33]. It is possible that idiosyncratic characteristics outweighed the typical attenuation effects of TC, reinforcing the need for case-by-case assessments, even with consistent general patterns.

Another important aspect refers to the difference between the perceptual data and the acoustic data analyzed. While in the research providing the database, perceptual judgments were made for 20-second segments, the acoustic data used here were extracted from the entire sample, which may seem to be a methodological discrepancy at first glance. However, this design was carefully planned. The VPA protocol used by the judges is widely validated in forensic phonetics because of its ability to capture a general overview of the speaker’s voice quality, even in relatively short excerpts. The 20-second segment showed sufficient variability in vocal emission to reflect predominant phonation patterns, as discussed in Gold and French (2011) [6] and Passetti and Constantini (2019) [23]. Therefore, the difference in length between the perceptual and acoustic data does not represent a bias, but rather an informed methodological choice.

Despite the promising findings, some limitations should be acknowledged. First, the sample size, although adequate for the exploratory purpose of this study, limits the statistical generalization of the results. Also, the study focused only on a subset of non-modal phonations (creak and creaky voice), as these were the only judgments with significant occurrence among the participants. Future analysis of other non-modal phonations could expand the understanding of the relationship between acoustic and perceptual data.

Despite that, the results obtained in this study offer significant contributions to the field of forensic phonetics. First, the study reinforces the importance of considering the effect of the recording channel in both acoustic analysis and perceptual interpretation of the voice. Compression and filtering of the telephone signal not only alter the spectral-temporal content of the sound but also directly impact the judgment of voice quality. Second, the findings show that, despite these limitations, some acoustic measurements remain capable of reflecting relevant perceptual patterns, which represents practical methodological implications for the forensic work. For example, the conscious selection of more stable measurements, such as jitter and HNR, can improve the reliability of acoustic analysis in forensic contexts where channel variation is present.

Finally, this study helps strengthen the empirical foundation required for forensic phonetics. By investigating the relationship between acoustic and perceptual data in different recording channels, this analysis fosters useful methodological reflections for experts, phoneticians, and other legal practitioners. The exploratory nature of the findings does not reduce their practical value; on the contrary, it shows paths for broader future studies while providing valuable interpretative tools for practical voice examination.

## Conclusion

This study results show a significant relationship between specific acoustic measurements and the perceptual judgment of non-modal phonations, despite the limitations of the telephone channel. Measurements such as jitter, HNR, spectral emphasis, intensity variation quotient, and f0 variations, were particularly sensitive to the vocal characteristics associated with creaky phonation and showed a correlation with perceptual scores even with degraded signals. At the same time, it became clear that compression and filtering of the telephone channel affect the extraction of several acoustic measurements and can mask relevant features of vocal emission.

Therefore, our study reinforces the importance of contextually informed analyses in forensic phonetics, with special attention to the technical recording conditions and the careful selection of the acoustic variables. Provided that it is conducted with clear methodological criteria, the integration of acoustic analysis and perceptual judgment can increase the robustness of the Speaker Comparison examination, contributing to more reliable interpretations in forensic contexts.
